# Genomic research perspectives in Kazakhstan

**DOI:** 10.5195/cajgh.2013.101

**Published:** 2014-01-24

**Authors:** Ainur Akilzhanova

**Affiliations:** Center for Life Sciences, Nazarbayev University, Astana, Kazakhstan

**Keywords:** genomic research, biomedical science, Kazakhstan

## Abstract

**Introduction:**

Technological advancements rapidly propel the field of genome research. Advances in genetics and genomics such as the sequence of the human genome, the human haplotype map, open access databases, cheaper genotyping and chemical genomics, have transformed basic and translational biomedical research. Several projects in the field of genomic and personalized medicine have been conducted at the Center for Life Sciences in Nazarbayev University. The prioritized areas of research include: genomics of multifactorial diseases, cancer genomics, bioinformatics, genetics of infectious diseases and population genomics. At present, DNA-based risk assessment for common complex diseases, application of molecular signatures for cancer diagnosis and prognosis, genome-guided therapy, and dose selection of therapeutic drugs are the important issues in personalized medicine.

**Results:**

To further develop genomic and biomedical projects at Center for Life Sciences, the development of bioinformatics research and infrastructure and the establishment of new collaborations in the field are essential.

Widespread use of genetic tools will allow the identification of diseases before the onset of clinical symptoms, the individualization of drug treatment, and could induce individual behavioral changes on the basis of calculated disease risk. However, many challenges remain for the successful translation of genomic knowledge and technologies into health advances, such as medicines and diagnostics.

It is important to integrate research and education in the fields of genomics, personalized medicine, and bioinformatics, which will be possible with opening of the new Medical Faculty at Nazarbayev University. People in practice and training need to be educated about the key concepts of genomics and engaged so they can effectively apply their knowledge in a matter that will bring the era of genomic medicine to patient care. This requires the development of well-equipped laboratories, bioinformatics, as well as qualified trained physicians and laboratory staff.

